# Immune responses to typhoid conjugate vaccine in a two dose schedule among Nepalese children <2 years of age

**DOI:** 10.1016/j.vaccine.2024.02.010

**Published:** 2024-02-22

**Authors:** Sanjeev M. Bijukchhe, Meeru Gurung, Bhishma Pokhrel, Mila Shakya, Dikshya Pant, Pratistha Maskey, Himang Maskey, Babita Dhakal, Shristy Rajkarkinar, Sabitri Bista, Merryn Voysey, Yama F. Mujadidi, Young Chan Kim, Rachel Atherton, Elizabeth Jones, Florence Mclean, Sonu Shrestha, Matilda Hill, Katherine Theiss Nyland, Sarah Kelly, Peter O’reilly, Ganesh Prasad Sah, Buddha Basnyat, Andrew J. Pollard, Shrijana Shrestha

**Affiliations:** a Deparment of Paediatrics, Patan Academy of Health Sciences, Kathmandu, Nepal; b Department of Paediatrics, University of Oxford, Oxford, United Kingdom; c Oxford University Clinical Research Unit, Patan Academy of Health Sciences, Kathmandu, Nepal; d NIHR Oxford Biomedical Research Centre, Oxford, United Kingdom

**Keywords:** Typhoid, Vaccine, Conjugate, Booster, Nepal

## Abstract

**Background:**

Previously, the Vi-typhoid conjugate vaccine (Vi-TT) was found to be highly efficacious in Nepalese children under 16 years of age. We assessed the immunogenicity of Vi-TT at 9 and 12 months of age and response to a booster dose at 15 months of age.

**Methods:**

Infants were recruited at Patan Hospital, Kathmandu and received an initial dose of Vi-TT at 9 or 12 months of age with a booster dose at 15 months of age. Blood was taken at four timepoints, and antibody titres were measured using a commercial ELISA kit. The primary study outcome was seroconversion (4-fold rise in antibody titre) of IgG one month after both the doses.

**Findings:**

Fifty children were recruited to each study group.Some visits were disrupted by the COVID19 pandemic and occurred out of protocol windows. Both the study groups attained 100 % IgG seroconversion after the initial dose. IgG seroconversion in the 9-month group was significantly higher than in the 12-month group (68.42 % vs 25.8 %, p < 0.001). Among individuals who attended visits per protocol, IgG seroconversion after the first dose occurred in 100 % of individuals (n = 27/27 in 9-month and n = 32/32 in 12-month group). However, sero-conversion rates after the second dose were 80 % in the 9-month and 0 % in the shorter dose-interval 12-month group (p < 0.001) (n = 16/20 and n = 0/8, respectively).

**Interpretation:**

Vi-TT is highly immunogenic at both 9 and 12 months of age. Stronger response to a booster in the 9-month group is likely due to the longer interval between doses.

## 1 Background

Typhoid fever is a major public health concern globally, with 9.24 million (95 % UI 5.94–14.1) cases in 2019, resulting in 110,000 deaths (95 % UI 52,800–191,000) [1]. Low- and middle-income countries (LMICs) are disproportionately affected. Nepal has one of the highest burdens of the disease, especially among children [2]. The true incidence of typhoid fever in many LMICs is unknown, as the reported numbers are likely to be an underestimate. Over the past few decades the emergence of increasingly antibiotic-resistant typhoid strains has been a significant issue [3–5]. Building an infrastructure for adequate water, sanitation, and hygiene practices in LMICs to eliminate typhoid fever may take decades. An effective vaccination program that concentrates on the highest-risk populations is likely to be the most beneficial and cost-effective control measure in these settings [6–8].

Typhoid conjugate vaccine (TCV) is advised for 6 months to 45 years of age by the World Health Organisation (WHO) [9]. The Gavi has been supporting the introduction of the TCV in national immunisation schedules and catch-up programmes for children up to 15 years old [10]. Currently, a tetanus toxoid conjugate vaccine (Vi-TT) and a diphtheria protein (cross-reacting material) conjugate vaccine (Vi-CRM197) are the only WHO pre-qualified typhoid conjugate vaccines [11]. Studies in different settings have demonstrated the safety and efficacy of the Vi-TT vaccine among children, including those under the age of two years, the likely target age group for use of the vaccine in the Expanded Programme on Immunization (EPI) schedules. However, the medium- and long-term efficacy of the vaccine remain unknown, and studies are currently underway to establish this [12]. These results will help inform whether booster doses will be required in countries where the TCV is in use. A sub-study of a large Vi-TT randomized controlled trial in India demonstrated a large post-booster rise in antibody titres in children and adults [13].

A large randomized controlled trial in Nepal demonstrated that a single dose of Vi-TT is immunogenic and effective in reducing S. Typhi bacteremia in Nepalese children aged nine months to under 16 years – however children under two were under-represented in the study [14]. We set up an additional prospective cohort study to answer an exploratory objective of the initial TyVAC trial to determine if the Vi-TT is immunogenic among Nepalese children at 9 or 12 months and whether there is a booster response to the second dose when given within a short interval, as a part of the Typhoid Vaccine Acceleration Consortium (TyVAC), with the aim of establishing the field efficacy of TCV. Among the different strategies for a vaccine’s immunogenicity, the time interval between priming and boosting is one that can affect the response to the vaccine [15]. Thus we aimed to assess the immunogenicity of Vi-TT in children aged 9 months and 12 months, as well as the response to a booster dose of Vi-TT given at 15 months in two subsets of children who received an initial dose at 9 or 12 months.

## 2 Research in context

### 2.1 Evidence before the study

We searched PubMed for research articles on the immunogenicity, efficacy, and effectiveness of typhoid conjugate vaccine in children published any time before October 2, 2022, with no language restriction. We used the search terms “typhoid fever”, AND “conjugate vaccine”, AND “children” AND/OR “booster”. Despite the Vi-rEPA showing more than 90 % efficacy in a double-blind, randomized control trial of children aged 2–5, the vaccine is not yet commercially available [16]. In a randomized controlled trial in India, Vi-TT was well-tolerated and produced robust and long-lasting serum anti-Vi IgG in children under two. A booster dose was administered two years after the initial vaccine and showed a strong response [13]. Likewise, the Vi-TT was reported to be effective in a case-control study involving children aged 6 months to 15 years in Pakistan [17,18]. Studies from Bangladesh and Malawi published in 2021also showcased the Vi-TT to be highly efficacious among children [18–20].

Previously, Shakya et al. also reported similar results from the interim analysis of a randomized control trial of Vi-TT in Nepalese children showing that a single dose of typhoid conjugate vaccine (TCV) given to children aged 9 months to younger than 16 years conferred over 80 % protection in the first 12 months after vaccination [14].

### 2.2 Added value of this study

This study shows the immunogenicity of the Vi-TT in children at 9 months and 12 months of age. Additionally, we report the response to a booster dose at 15 months in both the study groups. Our results showed substantial seroconversion after the primary dose at 9 months or 12 months of age; however, after the booster at 15 months, seroconversion in the 9-month group was higher than in the 12-month group.

### 2.3 Implications of all the available evidence

The study results show that Vi-TT vaccine induces robust immune responses at both 9 months and 12 months of age. However, children who received the initial dose at 9 months of age showed a stronger reaction to a booster, most likely as a result of the longer interval between the initial dose and booster in this group.

## 3 Methods

### 3.1 Study design and participants

A prospective cohort study was carried out, with participants recruited in two study groups of infants aged 9 and 12 months from Patan Hospital, Kathmandu. Participants were eligible for enrolment if they were of 9 months or 12 months age (age windows in Table 1), in good health, could comply with the study follow-up requirements, lived within the study catchment area, and if their parent/legal guardian provided informed consent. Any participant who was known to be allergic to any vaccine component, had a medical condition that prevented them from completing the study’s requirements, or had plans to leave the study catchment area within six months were excluded. If fever was identified, the participant was asked to return in >48 h following cessation of fever for re-affirmation of consent.

A written informed consent for enrolment was obtained from parents or legal guardians. At enrolment all participants had an initial dose of Vi-TT, which was followed by a booster dose at 15 months of age. Blood samples were collected immediately before and at one month after the first dose of vaccine and then, immediately before and one month after the booster dose. AEFI and SAE were followed-up passively and parents or guardians were invited to contact the study doctor if they observed any adverse events following vaccination or if their child had any significant health events or hospitalizations that occurred during the study period.

### 3.2 Vaccine (Intervention)

The participants were given initial and booster doses of tetanustoxoid conjugated Vi polysaccharide typhoid vaccine (Typbar TCV; Bharat Biotech International, India) of 25 μg/0⋅5 mL dosage. Details of the vaccine characteristics are presented in Supplemental Table 2.

### 3.3 Outcomes

The study outcome was the anti-Vi IgG antibody levels in blood samples collected one month (28 Days) after the booster dose of Vi-TT. Seroconversion was defined as the 4-fold increase in antibody titre.

### 3.4 Recruitment

Participants were recruited after assessing the inclusion and exclusion criteria, and receiving written informed consent. Basic medical history was taken, and temporary exclusion criteria were checked. Once consented, participant’s demographic information (including age and address) and contact details were collected. All details were recorded in an eCRF (REDCap).

### 3.5 Study groups and visits

At enrolment, at 9 or 12 months of age, all participants received an initial dose of 25 μg/0⋅5 mL of Vi-TT (Typbar TCV; Bharat Biotech International, India). At 15 months of age, both groups received a second dose of the same vaccine. Protocol windows for vaccination and follow-ups are presented in Supplemental Table 1.

### 3.6 Blood samples

Blood samples were planned at four timepoints; immediately before and 28 days after receiving each vaccine dose. Blood samples were transported to Patan Hospital Lab, where they were processed and stored by trained study staff and later shipped to Oxford University Laboratory for the analysis, in accordance with standard operating procedures.

Anti-Vi IgG and Anti-IgA titres were measured in plasma samples at the Oxford Vaccine Group Laboratory, University of Oxford. Anti-Vi IgG titres were measured employing a commercial ELISA kit (VaccZyme, The Binding Site, Birmingham, UK) according to the manufacturer’s guidelines. Anti-Vi IgA titres were assessed with Vi-coated plates and reagents supplied by The Binding Site using a protocol adapted from the commercial VaccZyme assay.

### 3.7 Statistical analysis

A target sample size of 100 children was required in the study, 50 each in 9-month and 12-month groups, under the assumptions of 80 % power, geometric means of 744 EU/ml and 372 EU/ml at the post-booster time point, and a SD on the log10 scale of 0.4804, and a 15 % drop out rate.

Due to the Covid-19 pandemic, recruitment and follow up was interrupted and many participants had to be vaccinated and followed up outside the protocol window. Hence, we did two analyses, the first, including all visits irrespective of the follow-up time, the second, including only the visits made within the protocol window. Analyses were conducted separately for the 9-month and 12-month age groups. Geometric mean concentration (95 % CI) and median (IQR, EU/mL of Anti-Vi IgG and Anti-Vi IgA for every visit were described. Fold-rise and the percentage with a 4-fold rise in antibody titres were calculated one month (28 Days) after the primary and booster doses of Vi-TT. T-test and Wilcoxon rank-sum test were used to compare groups. p-values less than 0⋅05 were considered significant. StataSE 17 was used for all the analyses.

This trial is registered with the ISRCTN registry, ISRCTN43385161. The study was reviewed and approved by the Oxford Tropical Research Ethics Committee, the Nepal Health Research Council and the Institutional Review Committee (IRC) Of Patan Academy of Health Sciences and was done in accordance with the principles of the Declaration of Helsinki.

## 4 Results

### 4.1 Participants

From 22nd December 2019 to 19th April 2021, a total of 100 healthy participants were recruited – 50 each aged 9 months and 12 months. The proportions of male were 62 % and 58 % in the 9-month and 12-month groups, respectively (Table 1). Following initial vaccination, there were 4 AEFIs, and one SAE (febrile seizure) which was considered unrelated to the vaccine by the study paediatrician. There were no AEFIs or SAEs reported following the booster vaccination.

### 4.2 Study visits

Due to the Covid pandemic, 86 of the 223 follow-ups were performed outside of the protocol windows, including two unsuccessful blood draws that were not included in the analysis (Supplemental Fig. 1).

The intervals between the first vaccination (visit 1) and booster visit (visit 3) in the 9- and 12-months were 191.5(178–244) and 199(78–307) median days (IQR), respectively (Table 1). However, when restricted to participants who made visits as defined by the protocol, the intervals between visit 1 and visit 3 in the 9- and 12-month groups were 182.5 (175–196) and 78 (72–87) median days (IQR), respectively (Table 1). 32 of the 43 participants in the 9-month group and 16 of the 37 participants in the 12-month group received their second dose within the protocol defined window.

The study did not proceed as anticipated due to the covid pandemic, and 84 of 321 blood samples were taken outside the protocol defined window, which may have led to bias. A total of 169 and 152 blood samples from 9-month and 12-month participants, respectively, were collected for analysis. Of these, 129 and 108 blood samples from 9-month and 12-month participants, respectively, were collected within the windows specified by the protocol (Supplemental Fig. 1).

### 4.3 Immunogenicity after the primary dose at 9 or 12 months of age

#### 4.3.1 All participants

In all participants of both age groups, IgG titre increased after primary Vi-TT immunisation, meeting the criteria for seroconversion at 28 days. Antibody levels waned thereafter but persisted well above baseline levels up to 15 months of age. The IgG level in the 9-month group increased from a GMC of 4.27 (95 % CI 3.60–5.06) EU/ml at baseline to 2280.59 (95 % CI 1670.71 to 3113.11) EU/ml at 28 days post-primary; and in the 12-month group from 4.30 (95 % CI 3.67–5.04) EU/ml to 2533.10 (95 % CI 2069.10–3101.15) EU/ml at 28 days post-primary. Likewise, IgA levels rose modestly in the 9-month group from a GMC of 1.66 (95 % CI 1.54–1.77) EU/ml at baseline to 35.20 (95 % CI 24.67–50.23) EU/ml at 28 days post-primary; and in the 12-month group from 1.83 (95 % CI 1.58 to 2.11) EU/ml to 48.96 (95 % CI 37.02–64.73) EU/ml at 28 days post-primary. All participants aside from one 12-month participant had IgA seroconversion after the first dose; 67.4 % in the 9-month group and 70.3 % in the 12-month group achieved IgA seroconversion at the time of booster (see Supplemental Figs. 2 and 3).

#### 4.3.2 Visits occurring per protocol windows

**Fig. 1:**
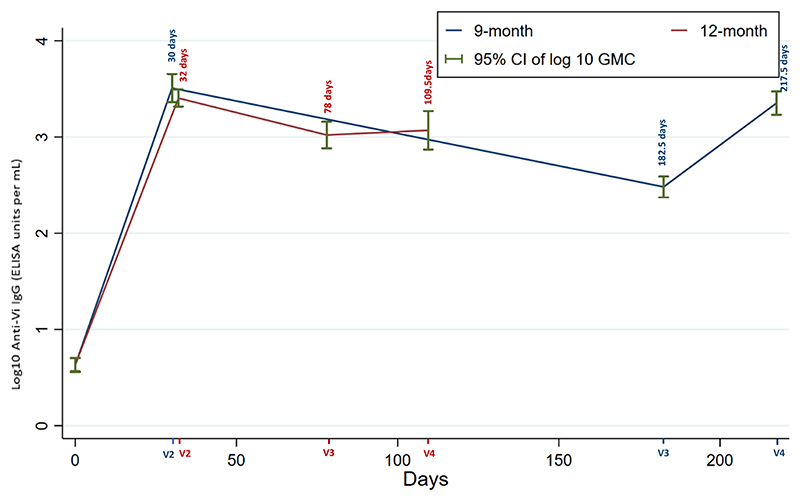
In participants who made per protocol visits, a line graph showing the geometric mean IgG (95 % CI) of 9-month and 12-month groups in median days of the four visits (9-month group visits: v2 = 30 days, v3 = 182.5, and v4 = 217.5; 12-month group visits: v2 = 32 days, v3 = 78 days and v4 = 109.5 days).

**Fig. 2:**
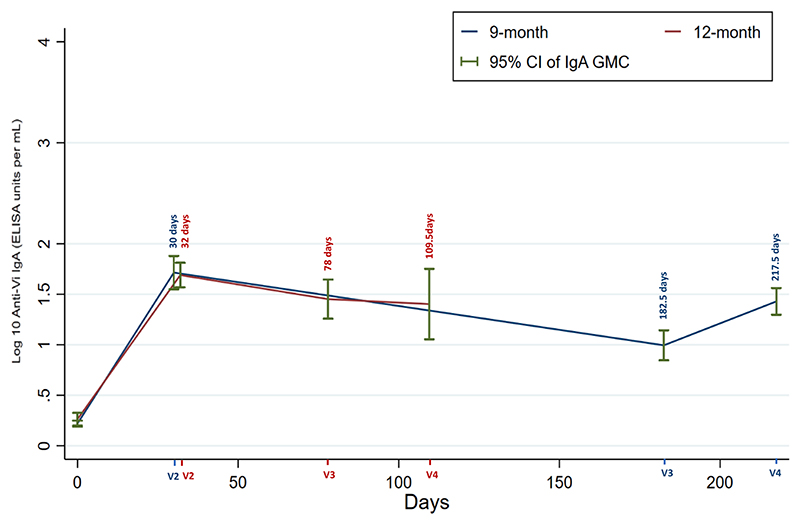
In participants who made per protocol visits, a line graph showing the geometric mean IgA (95 % CI) of 9-month and 12-month groups. in median days of the four visits (9-month group visits: v2 == 30 days, v3 = 182.5, and v4 = 217.5 days; 12-month group visits: v2 = 32 days, v3 = 78 days and v4 = 109.5 days).

When analysis was restricted to only those who attended visits per protocol, IgG levels increased from a GMC of 4.27 (95 % CI 3.60–5.06) EU/ml at baseline to 3223.51 (95 % CI 2302.10–4513.71) EU/ml at 28 days post-primary; and from 4.30 (95 % CI 3.67–5.04) EU/ml at baseline to 2553.10 (95 % CI 2069.10–3101.15) EU/ml at 28 days post-primary in the 9-month and 12-month groups, respectively. In all participants in both age groups, IgG levels increased after primary Vi-TT immunisation, meeting the criteria for seroconversion at 28 days. Similarly, IgA levels rose from a GMC of 1.66 (95 % CI 1.54–1.77) EU/ml at baseline to 51.80 (95 % CI 35.42–75.75) EU/ml at 28 days post-primary, and 1.83 (95 % CI 1.58–2.11) EU/ml at baseline to 48.96 (95 % CI 37.02–64.73) EU/ml at post-primary in the 9-month and 12-month groups respectively. All the 9- and 12-month groups subjects showed IgA seroconversion at 28 days post-primary. At the time of booster, 75 % of the 9-month group and 87.5 % of the 12-month group had sustained seroconversion (see Tables 2, 3, and Figs. 1, 2).

### 4.4 Immunogenicity after the booster at 15 months of age

#### 4.4.1 All visits

In both groups, the IgG titre level increased after the booster dose, however, the increase was less than after the primary dose. In comparison with the 12-month individuals, IgG seroconversion immediately before to 28 days after the booster was significantly higher in the 9-month group (68.42 % vs 25.8 %). When the baseline level was compared with the post-booster level, all but a few participants demonstrated seroconversion.

The IgA response to the booster was less pronounced than the IgG response. The 9-month group had a higher seroconversion rate than the 12-month group, however, the majority of participants had maintained 4-fold increase when the post-booster level was compared with baseline (see Supplemental Figs. 2 and 3).

#### 4.4.2 Visits made per protocol windows

Only the 9-month group showed a significant rise in IgG titre after the booster (GMC 2249.26, 95 % CI 1701.02–2974.19), however, the titre was still lower than after the primary response; the 12-month group showed only a modest increase (GMC 1173.43, 95 % CI 740.17–1860.31). A significant difference in IgG seroconversion was seen between the two age groups; in the 9-month group, 80 % (n = 16/20) participants demonstrated a four-fold rise in antibody titre, compared with none (n = 0/8 0 %) in the 12-month group. However, when compared with the pre-vaccination baseline titres, all participants of both the groups had demonstrable seroconversion after the booster dose. For IgA, only the 9-month group showed an increase in GMC titre (GMC 26.87, 95 % CI 19.84–36.39). There was no IgA seroconversion in the 12-month group (n = 0/8), whereas in the 9-month group almost half (n = 9/20) of the participants seroconverted. Compared with the baseline levels, IgA seroconversion was sustained in all but two of the 12-month subjects (see Tables 2, 3 and Figs. 1, 2).

## 5 Discussion

Previously, Shakya and colleagues reported the immunogenicity and efficacy from a randomized controlled trial of Vi-TT, the TyVAC trial; in which, children under two years of age were under-represented [14]. Here, we report immunogenicity results in Nepalese children at both 9 months and 12 months of age and a booster at 15 months of age. We found the Vi-TT to be highly immunogenic at both 9 and 12 months of ages with sharp increments observed in both IgG and IgA levels after the primary dose. After the booster dose, we also observed a rise in the antibody titres; however, the magnitude of the rise in both the groups was modest compared with the increases after the primary dose.The 4-fold rise (seroconversion) in IgG titre in the 9-month group was 68.42 %, after the booster dose - however only 25.8 % seroconverted in the 12-month group which perhaps reflect the shorter interval between primary dose and booster dose in this group, resulting in higher pre-boost anti-body levels and greater inhibition of antibody responses to the booster dose.

Due to the interruption in enrolment and follow-up caused by the Covid19 pandemic, an additional analysis was conducted including only the visits made within the protocol windows. For the primary dose, we observed 100 % IgG seroconversion in both the groups. However, for the booster dose, there was a difference between the groups with 80 % IgG seroconversion in the 9-month group and no seroconversion among the 12-month participants observed. Our results in this subset analysis showed that a booster after a wider interval may generate a better boost of antibodies, although some potential confounding by the ages at which the first dosages were administered cannot be ruled out.

The findings of our investigation validate the immunogenicity data of Vi-TT in young children as previously reported by several investigators. In a prelicensure phase 3 trial of the Vi-TT in India by Mohan et al, the Vi-TT was found to be highly immunogenic in the open label trial with 98.1 % (95 % CI, 95.7–99.2) seroconversion at day 42 after a first dose immunization observed among 307 children aged 6–23 months and in the controlled trial, the Vi-TT attained higher seroconversion rate than the Typbar Vi-polysaccharide vaccine [13]. The study also assessed responses to a booster dose, the findings of which were consistent with the robust booster immune response in our study’s 9-month group, which did not, however, exceed the post-primary level [21]. The trial however, administered a booster dose at a 24 month interval, as opposed to the 3 and 6 months interval in our study. Later follow up demonstrated only a modest difference in seroconversion between the boosted group and a non-boosted group at 3 and 5 years [21].

There is already good evidence on the efficacy and effectiveness of the Vi-TT from large scale studies in Malawi, Bangladesh, Nepal and Pakistan. However, these studies have involved a single dose regimen with short follow-up duration to-date. A phase 3, double blind trial from Malawi found that Vi-TCV had an efficacy rate of 74.4 % (31.7–90.4) among children of 9 months to five years of age [19]. In Bangladesh, Qadri et al. reported vaccine protection of 81 % (95 % CI 39–94) among toddlers of 9 months to two years of age and a total Vi-TCV protection of 85 %,(95 % CI 76–91) among children aged 9 months to 16 years old. The anti-Vi IgG responses were robust in Vi-TT recipients of all age groups [20]. Likewise, in Pakistan, vaccine effectiveness of 72 % (95 % CI: 34 % 88 %) was reported among children aged 6 months to 15 years [17,20]. Recently, 98 % vaccine effectiveness was reported in Pakistan in a field evaluation of the catchup campaign before the introduction of VI-TT in the country’s routine vaccination [22].

Booster doses are often advised for many vaccines. Both the primary dose, which establishes the host immune response, and the booster dose, which amplifies and focuses the initial reaction, may be important [23]. The interval between a primary and a booster dose has been shown to influence the post-second dose response, and has been highlighted recently for COVID-19 vaccines [24]. Taranger et al. also presented evidence on the significance of prolonging the time between doses, suggesting that a longer interval increases the immunogenicity of vaccines given to infants [15]. Our findings also suggested that extending the prime-boost interval could improve Vi-immune TCV responsiveness, which could be linked to inadequate B cell maturation and greater antibody inhibition in shorter prime-boost intervals [25]. Recent evidence suggests vaccine doses can be mixed and matched. The Quebec Immunisation Committee endorsed a mixed schedule for PCV in 2017 in favor of its immunogenicity, protection against otitis media, pneumonia and IPD, safety, herd immunity, acceptance, cost-effectiveness, feasibility, and compliance [26,27]. A meningococcal group C(MenC) vaccination trial found that infants who received tetanus toxoid conjugated MCC(MCC-TT) vaccine followed by cross reacting material conjugated MCC (MCC-CRM) had adequate protection - however, MCC-CRM followed by MCC-TT showed significantly lower MenC and Hib antibody responses. Understanding if the ViTT TCV and Vi-CRM197 TCV are interchangeable for heterologous boosting will provide vaccine policymakers more options for immunization schedules [28]. A limitation is the small sample size because of the exclusions, particularly in the 12-month group.

The key issues are whether a Vi-TT booster dose is necessary, which will be determined by ongoing studies on the duration of vaccine efficacy and, if so, when it should be given. The WHO has recommended the immunization of children under two years of age in the Expanded Programme on Immunization schedule to promote immunity that would last until school-age [29]. There is evidence that children aged 6–23 months are responsive to Vi-TT [13,21]. A booster dose, however, might defend against waning of protection, particularly during the period of greatest risk during primary school [23]. According to our data, the immune response was greater in the 9-month group with a longer primary-booster interval compared with the 12-month group with a shorter primary-booster interval. A booster dose prior to entering school may be appropriate, as shown by the Indian booster trial showing durability of antibody even five years following Vi-TT priming [21].

The results of our study provide additional assurances on the immunogenicity of the Vi-TT with high seroconversion to a single dose at both 9 and 12 months of age. If it is found that a second dose is needed, it may be best administered after a long interval and preferably before the period of heightened risk after school entry.

## Supplementary Material

Suppl

## Figures and Tables

**Table 1:** General characteristics of all participants and after excluding out-of-the-window participants in 9-month and 12-month groups. p-values are based on Wilcoxon rank-sum test.FU = Follow-up; PW = protocol windows.

Characteristics	9-month	12-month	p-value
*All participants*			
Mean Age in months (SD)	9.36 (0.42)	12.63(0.45)	
Sex (male %)	62 %	58 %	0.564
*Visit Intervals*			
Visit 1–2 Interval (days)	34.5 (29–67)	32 (30.5–39)	0.7229
Median (IQR)Permissible time	(21–49 days)	(21–49 days)	
Visit 1–3 Interval (days)	191.5 (178–244)	199 (78–307)	
Median (IQR)Permissible time	(168–241 days)	(77–150 days)	
Visit 3–4 Interval(days)	35 (32–49)	35 (32–66)	0.6274
Median (IQR)Permissible time	(21–49 days)	(21–49 days)	
*Visits made as per Protocol Windows*			
Proportions of visits made within PW			
Visit 1 (FUs per PWs/ Total FUs)	50/50	50/50	
Visit 2(FUs per PWs/ Total FUs)	27/38	32/32	
Visit 3 (FUs per PWs/ Total FUs)	32/43	16/37	
Visit 4 (FUs per PWs/ Total FUs)	20/38	10/33	
*Visit Intervals*			
Visit 1–2 Interval (days)	30 (29–35)	32 (30.5–39)	0.0257
Median (IQR)Permissible time	(21–49 days)	(21–49 days)	
Visit 1–3 Interval (days)	182.5 (175–196)	78 (72–87)	
Median (IQR)Permissible time	(168–241 days)	(77–150 days)	
Visit 3–4 Interval(days)	35 (27–37)	31.5 (28–35)	0.8318
Median (IQR)Permissible time	(21–49 days)	(21–49 days)	

**Table 2:** In participants who made as per protocol visits, IgG and IgA titres in 9-month and 12-month groups.

	9-month					12-month			
	Visit 1 Primary dose	Visit2 28 days post-primary	Visit 3 Booster dose	Visit 4 28 days Post-booster		Visit 1 Primary dose	Visit2 28 days post-primary	Visit 3 Booster dose	Visit 4 28 days Post-booster
*IgG*									
Level above the lower limit of quantification of the assay	4/50 (8 %)	27/27 (100 %)	32/32 (100 %)	20/20 (100 %)		5/50 (10 %)	32/32 (100 %)	16/16 (100 %)	10/10 (100 %)
Geometric mean concentration (95 % CI), EU per mL	4.27 (3.60–5.06)	3223.51 (2302.10–4513.71)	302.848 (235.845–388.89)	2249.26 (1701.02–2974.19)		4.30 (3.67to5.04)	2533.10 (2069.10–3101.15)	1049.01 (762.07–1443.99)	1173.43 (740.17–1860.31)
Median (IQR)	3.7 (3.7–3.7)	2854.66 (1871.47–6023.04)	332.1115 (174.97–472.62)	2151.273 (1396.04 to 3029.45)		3.7 (3.7–3.7)	2278.40 (1725.07–4147.27)	918.21 (727.17–1501.6)	1745.12 (1099.2–2492.64)
*IgA*									
Level above the lower limit of quantification of the assay	3/50 (6 %)	27/27 (100 %)	28/32 (87.5 %)	20/20 (100 %)		6/50 (12 %)	32/32 (100 %)	16/16 (100 %)	9/10 (90 %)
Geometric mean concentration (95 % CI), EU per mL	1.66 (1.54–1.77)	51.80 (35.42–75.75)	9.88 (7.01–13.92)	26.87 (19.84– 36.39)		1.83 (1.58–2.11)	48.96 (37.02–64.73)	28.31 (18.11–44.25)	25.29 (11.32–56.52)
Median (IQR)	1.56 (1.56 to1.56)	56.74 (28.09–77.06)	10.06 (6.20–19.68)	24.96 (18.44–45.56)		1.56 (1.56 to1.56)	46.51 (29.75–69.52)	25.26 (18.20–43.92)	31.71 (24.79–42.97)

**Table 3:** In participants who made as per protocol visits, Geometric mean fold rise(95% CI) and seroconversion of IgG and IgA in 9-month and 12-month groups.

	9-month					12-month			
	Visit 1–2	Visit 1–3	Visit 3–4	Visit 1–4		Visit 1–2	Visit 1–3	Visit 3–4	Visit 1–4
*IgG*									
Geometric mean fold rise (95 % CI)	1087.12 (616.49–1557.75)	87.90 (64.19–111.61)	10.03 (5.99–14.07)	684.72 (420.19to 949.24)		713.43 (544.21–882.65)	309.10 (186.41 to 431.79)	1.15 (0.73–1.57)	309.10 (186.41–431.79)
4-fold increase (%)	27/27 (100 %)	32/32 (100 %)	16/20 (80 %)	20/20 (100 %)		32/32 (100 %)	16/16 (100 %)	0/8 (0 %)	10/10 (100 %)
*IgA*									
Geometric mean fold rise (95 % CI)	51.57 (18.02 85.13)	9.59 (5.52–13.66)	4.26 (2.77–5.75)	21.41 (13.47–29.34)		100.61 (–26.20–227.43)	22.36 (11.46–33.26)	0.97 (0.65 to1.29)	22.83 (8.04–37.62)
4-fold increase (%)	27/27 (100 %)	24/32 (75 %)	9/20 (45 %)	20/20 (100 %)		31/32 (96.88 %)	14/16 (87.5 %)	0/8 (0 %)	8/10 (80 %)

## Data Availability

De-identified individual participant data from this study is available to researchers whose proposed use of the data has been approved after a signed data access agreement. Data can be obtained by contacting merryn.voysey@paediatrics.ox.ac.uk.
