# Supplementary material for: Immune responses to typhoid conjugate vaccine in a two dose schedule among Nepalese children <2 years of age
Source: Vaccine. Author manuscript; Available in PMC 2024 Sep 24. (PMC7616634; doi:10.1016/j.vaccine.2024.02.010)
Supplement: Suppl [file EMS198674-supplement-Suppl.docx]

**Supplemental materials**

|  | **Visit 1 (enrolment)**  9/12 months of age  ***Blood+Primary dose*** | **Visit 2**  28 days post-primary  ***Blood*** | **Visit 3**  15 months of age  ***Blood+booster*** | **Visit 4**  28 days post-booster  ***Blood*** |
| --- | --- | --- | --- | --- |
| **Protocol windows** | -2 weeks  /+1 month 29 days | -7  /+21 days | -2 weeks  /+1 month 29 days | -7  /+21 days |

Supplemental Table 1. Protocol windows for the study visits


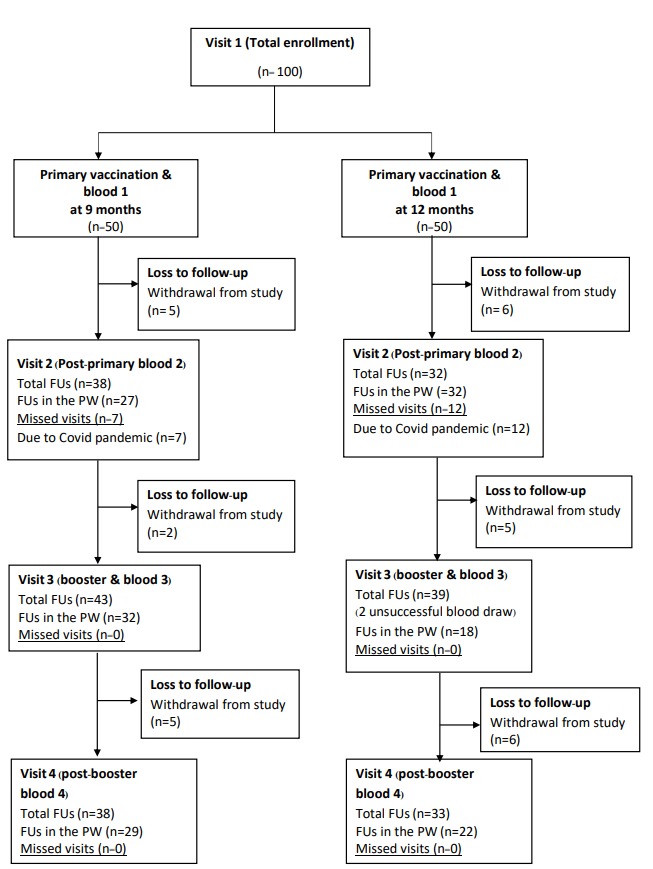
Supplemental Supplemental Fig.1 Flowchart showing follow-up (FU) numbers and FU visits made per Protocol windows (PW). Missed visits = FU visits missed but not withdrawn from the study.


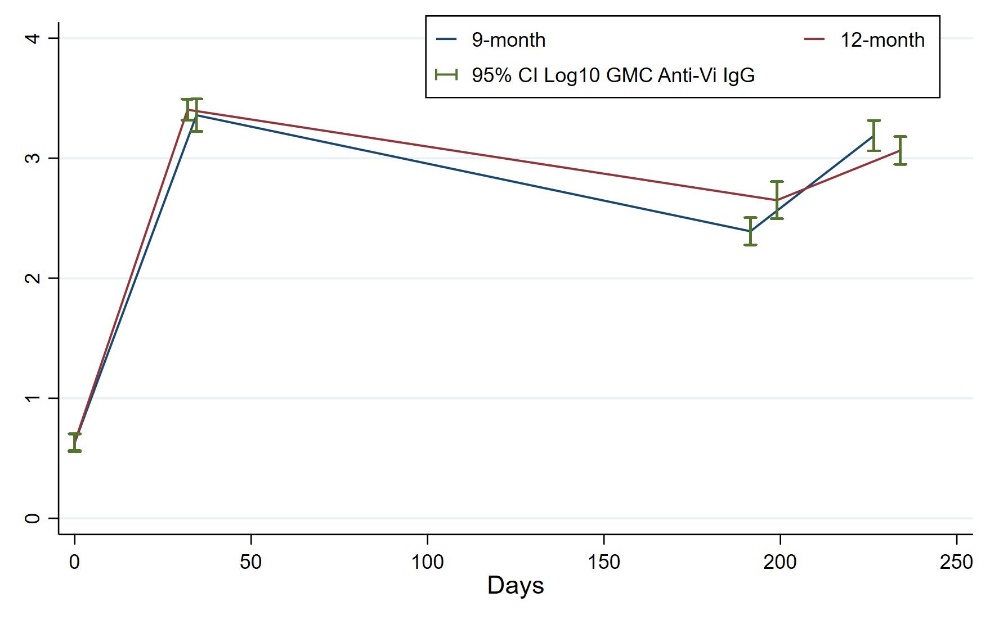


Log10 Anti-Vi IgG (ELISA units per mL)

Supplemental Figure 2. In all participants, line graph showing geometric mean IgG of 9-month and

12-month groups in median days of the four visits.


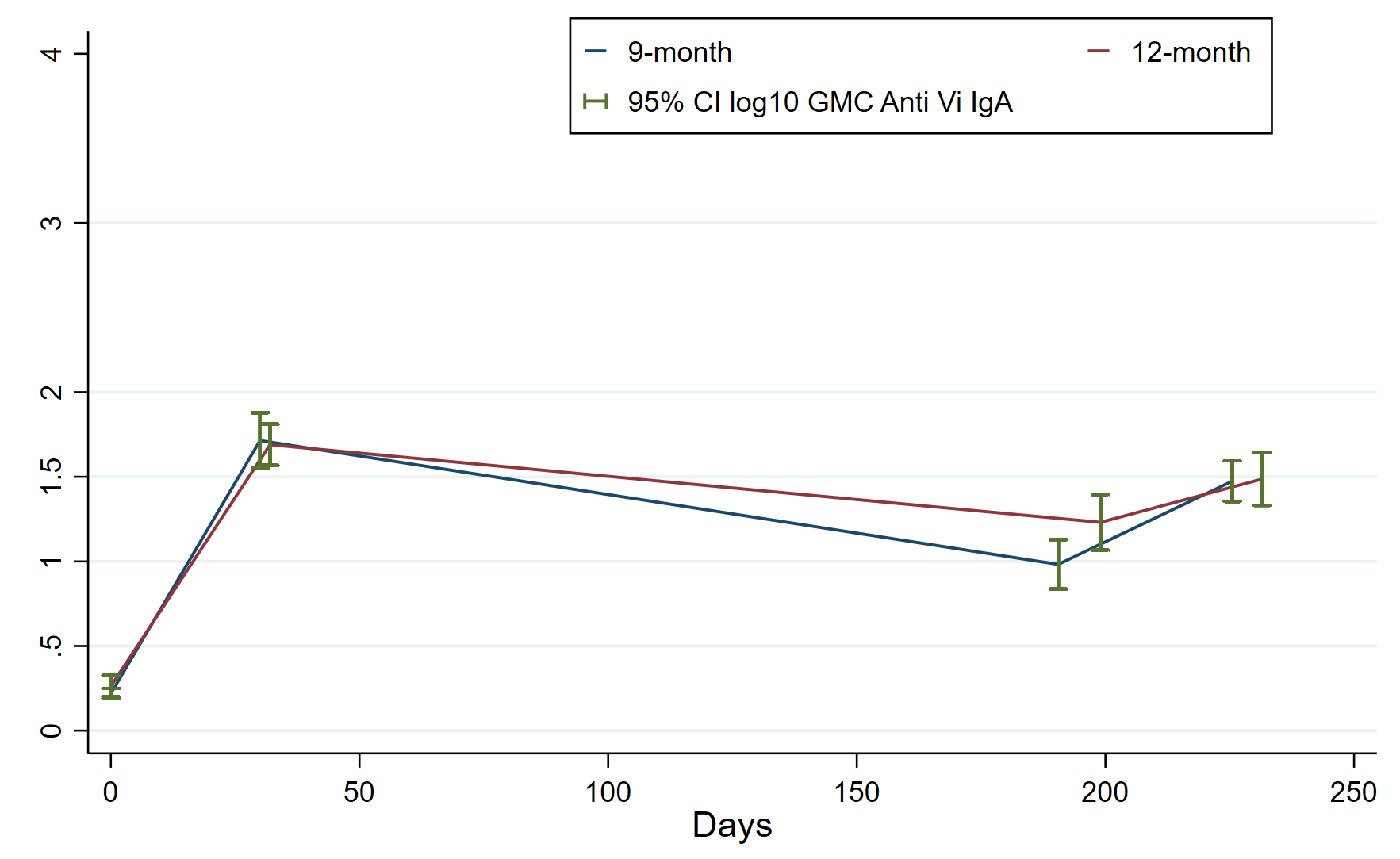


Log10 Anti-Vi IgA (ELISA units per mL)

Supplemental figure 3. In all participants, line graph showing geometric mean IgA of 9-month and 12-month groups in median days of the four visits.


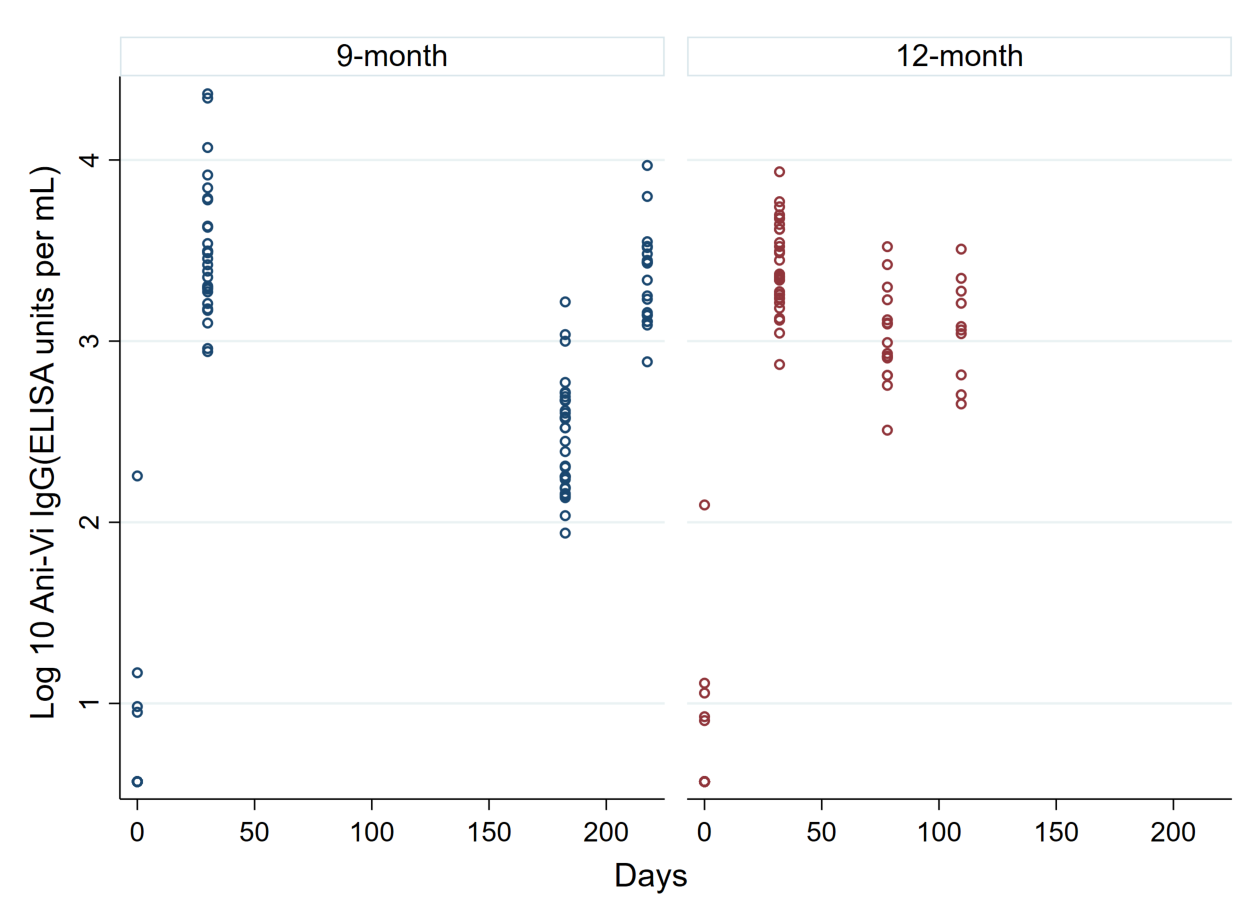


Supplemental Figure 4. In participants who made per protocol visits, a scatter graph showing the geometric mean IgG (95% CI) of 9-month and 12-month groups in median days of the four visits (9-month group visits: visit 2 == 30 days, visit 3 = 182.5 days,and visit 4= 217.5 days; 12-month group visits: visit 2=32 days, visit 3 = 78 days and visit 4= 109.5 days).


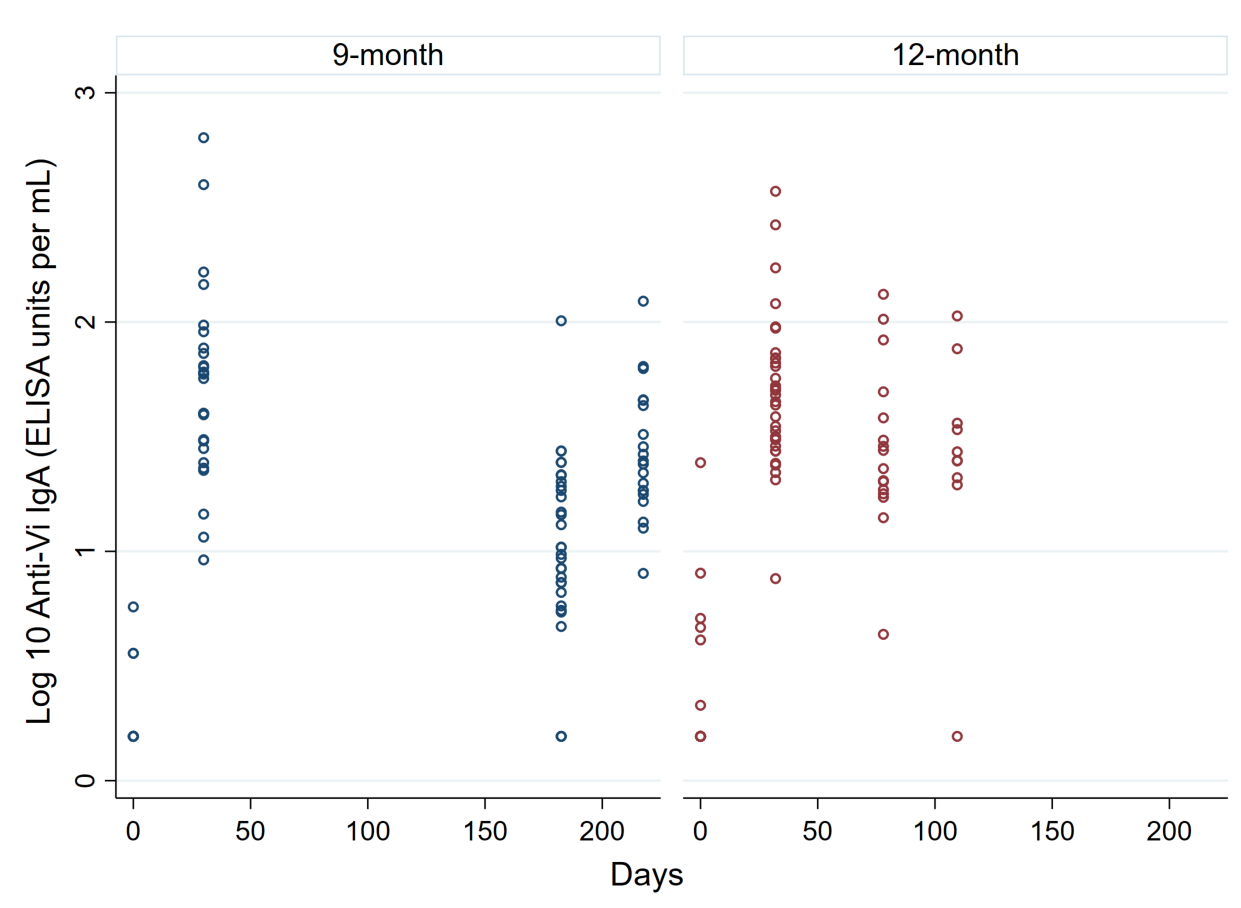


Supplemental Figure 5. In participants who made per protocol visits, a scatter graph showing the geometric mean IgA (95% CI) of 9-month and 12-month groups in median days of the four visits (9-month group visits: visit 2 == 30 days, visit 3 = 182.5,and visit 4= 217.5 days; 12-month group visits: visit 2=32 days, visit 3 = 78 days and visit 4= 109.5 days).

| Vaccine characteristics |
| --- |
| - Name of the vaccine: Tetanus-toxoid conjugated Vi polysaccharide typhoid vaccine (Typbar TCV; Bharat Biotech International, India) - Dosage: 25 μg/0·5 mL - Needle used for injection: 23 G - Cold chain maintained at: 2-8 degree celsius - Vaccine Lot numbers used: 76CJ17002 (exp 07/2020)   76CJ17004 (exp 08/2020)  76B18009A (exp 08/2021)  76C20043A (exp 06/2023) |

Supplemental Table 2: Vaccines used in the study and their characteristics. ROA= route of administration.
